# Pharmacological targeting of BET proteins inhibits renal fibroblast activation and alleviates renal fibrosis

**DOI:** 10.18632/oncotarget.12498

**Published:** 2016-10-06

**Authors:** Chongxiang Xiong, Monica V. Masucci, Xiaoxu Zhou, Na Liu, Xiujuan Zang, Evelyn Tolbert, Ting C. Zhao, Shougang Zhuang

**Affiliations:** ^1^ Department of Nephrology, Shanghai East Hospital, Tongji University School of Medicine, Shanghai, China; ^2^ Department of Medicine, Rhode Island Hospital and Alpert Medical School, Brown University, Providence, RI, USA; ^3^ Department of Nephrology, Shanghai Songjiang District Central Hospital, Shanghai, China; ^4^ Department of Surgery, Roger Williams Medical Center, Boston University, Providence, RI, USA

**Keywords:** bromodomain and extra-terminal proteins, unilateral ureteral obstruction, renal fibrosis, I-BET151, extracellular matrix proteins, Pathology Section

## Abstract

Bromodomain and extra-terminal (BET) protein inhibitors have been shown to effectively inhibit tumorgenesis and ameliorate pulmonary fibrosis by targeting bromodomain proteins that bind acetylated chromatin markers. However, their pharmacological effects in renal fibrosis remain unclear. In this study, we examined the effect of I-BET151, a selective and potent BET inhibitor, on renal fibroblast activation and renal fibrosis. In cultured renal interstitial fibroblasts, exposure of cells to I-BET151, or silencing of bromodoma in-containing protein 4 (Brd4), a key BET protein isoform, significantly reduced their activation as indicated by decreased expression of α-smooth muscle actin, collagen 1 and fibronectin. In a murine model of renal fibrosis induced by unilateral ureteral obstruction (UUO), administration of I-BET151 suppressed the deposition of extracellular matrix proteins, renal fibroblast activation and macrophage infiltration. Mechanistically, I-BET151 treatment abrogated UUO-induced phosphorylation of epidermal growth factor receptor and platelet growth factor receptor-β. It also inhibited the activation of Smad-3, STAT3 and NF-κB pathways, as well as the expression of c-Myc and P53 transcription factors in the kidney. Moreover, BET inhibition resulted in the reduction of renal epithelial cells arrested at the G2/M phase of cell cycle after UUO injury. Finally, injury to the kidney up-regulated Brd4, and I-BET151 treatment abrogated its expression. Brd4 was also highly expressed in human fibrotic kidneys. These data indicate that BET proteins are implicated in the regulation of signaling pathways and transcription factors associated with renal fibrogenesis, and suggest that pharmacological inhibition of BET proteins could be a potential treatment for renal fibrosis.

## INTRODUCTION

Fibrotic diseases contribute to ~45% of mortalities in developed countries [1] and represent a significant burden on public health systems. Chronic kidney disease (CKD) is a chronic fibrotic disease prevalent amongst roughly 10% of the world's population. In some cases, CKD will progress to the end stage of renal disease that necessitates replacement therapy or kidney transplantation [2, 3]. Currently, there is no available treatment to halt the progression of CKD despite evidence showing that of the renin-have been able to slow down this process in a certain population of patients [4].Therefore, it is important to develop new approaches that can deliver significant efficacy in treating CKD.

Recently, epigenetics-based therapy has become the focus of scientific investigation. This approach involves the use of drugs or other techniques to treat medical conditions by targeting epigenetic mechanisms. Epigenetic regulation affects chromatin dynamics and nucleosome assembly. One such modification is histone acetylation which relaxes chromatin structure by loosening their interaction with DNA, thereby facilitating gene expression. However, the functional role of acetylated histones is regulated by bromodomains and extra-terminal (BET) domain family proteins [5]. It has been reported that members of BET proteins such as Brd2, Brd3, Brd4 are associated with acetylated chromatin and facilitate transcriptional activation through increasing the effective molarity of recruited transcriptional activators [6]pharmacological inhibition of bromodomain proteins that bind acetylated chromatin marks may repress downstream gene expression. Recently, a number of small-molecule inhibitors targeting bromodomain proteins such as JQ1 and I-BET151 have been developed [7–9]. These small molecules can bind to bromodomains of BET family proteins and compete with acetylated-lysine histone peptides [10]. The wealth of pre-clinical data supports BET inhibition as a promising new therapeutic strategy against cancer, inflammation and cardiovascular disease [11].

Recently, the efficacy of BET inhibitors has also been tested in animal models of tissue fibrosis. For example, Budd et al demonstrated that inhibition of Brd4 by JQ1 can block platelet derived growth factor (PDGF) and transforming growth factor-β1 (TGF-β 1)-induced migration and proliferation of lung fibroblasts isolated from patients with rapidly progressing idiopathic pulmonary fibrosis [12]. Oral administration of JQ1 was also capable of preventing the development of experimentally induced pulmonary fibrosis in mice [12]. In addition, Ding et al found that JQ1 was also effective in blocking activation and proliferation of hepatic stellate cell activation *in vitro* and *in vivo* [1]. Furthermore, in a carbon tetrachloride -induced mouse model of liver fibrosis, BET inhibitors were shown to prevent liver injury and reverse the progression of existing fibrosis [1]. Cistromic analyses indicated that BRD4 is co-localized with profibrotic transcription factors and concentrates at specific enhancers that are associated with genes involved in multiple profibrotic pathways [1]. A very recent study shows that inhibition of BET protein with JQ1 can ameliorate renal damage *via* suppressing renal inflammation [13]. To date, there are still no reports assessing the pharmacological effect of BET inhibitors on renal fibrosis. Like other chronic fibrotic diseases, CKD is characterized by the activation of fibroblasts and deposition of excessive amounts of extracellular matrix (ECM)proteins [3]. Renal fibroblast activation can be induced by the activation of multiple growth factor/cytokine receptors, such as TGF-β1 receptors, platelet derived growth factor receptors (PDGFR) and epidermal growth factor receptors (EGFR) [14]. The signals initiated from the receptors are then transduced by several intracellular signaling pathways, including Smad-3, signal transducer and activator of transcription 3 (STAT3), and nuclear factor-κB (NF-κB). The profibrotic growth factors/cytokines can be produced from renal tubular cells after injury [15]. Severely injured renal tubular cells usually undergo maladaptive processes and differentiate into a profibrotic phenotype characterized by G2/M arrest. These cells acquire an ability to produce and release excessive amounts of profibrotic factors, leading to renal interstitial fibroblast activation and fibrosis [16, 17]. It has been documented that many signaling molecules and transcriptional factors involved in renal fibrogenesis are subjected to epigenetic regulations, in particular, acetylation [18–20].Thus, the BET domain family of proteins may act as potent drivers of the fibrotic responses in the kidney after injury.

In this study, we examined the effect of BET protein inhibition on the activation of renal interstitial fibroblasts in cultured rat renal interstitial fibroblasts, as well as the development of renal fibrosis a murine model of renal fibrosis induced by unilateral ureteral obstruction by using I-BET151, a small molecule with potent binding affinity to BRD2, BRD3 and BRD4 [21].

## RESULTS

### I-BET151 inhibits activation and proliferation of renal interstitial fibroblasts

**Figure 1:**
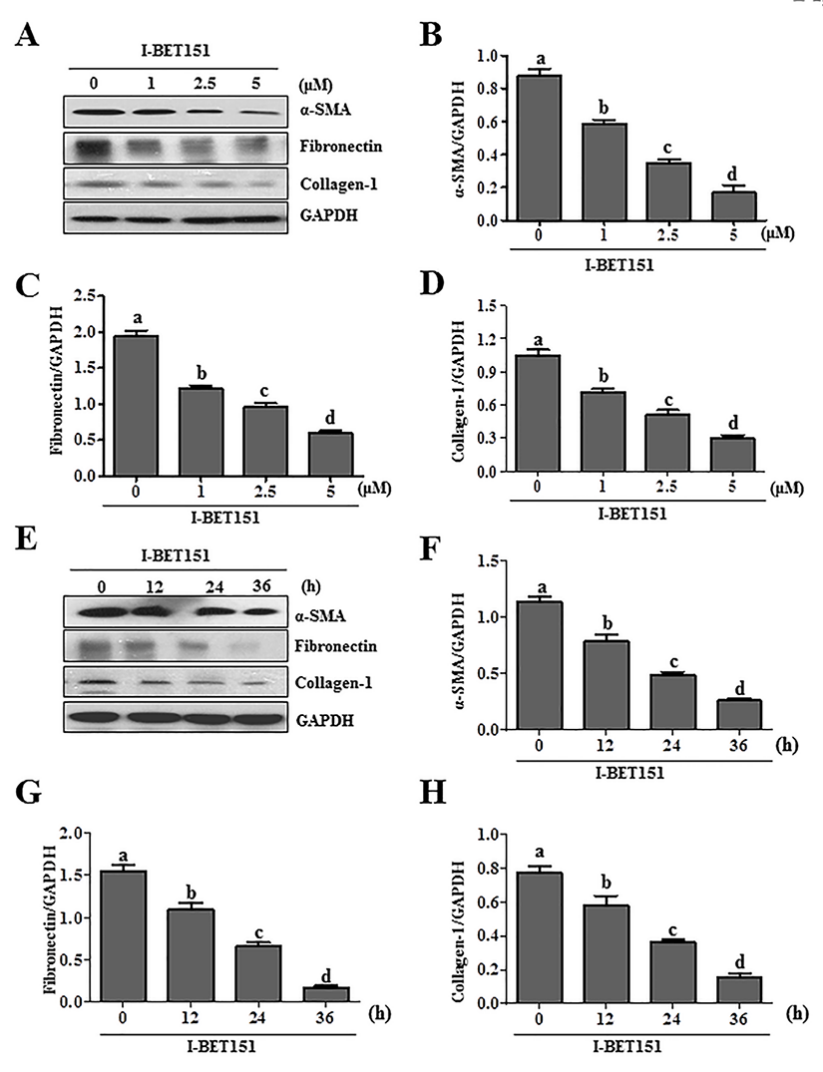
I-BET151 inhibits serum-induced activation of renal interstitial fibroblasts in a dose and time dependent manner Normally cultured NRK-49F cells were treated with I-BET151 (0-5μM) for 36h**A.**, or 5μM for (0-36h)**E.**. Then, cell lysates were prepared and subjected to immunoblot analysis with antibodies against α-SMA, collagen-1, fibronectin, and GAPDH **A.** and **E.** The levels of α-SMA **B.**, **F.**, fibronectin **C.**, **G.**, collagen-1 **D.**, **H.** were quantified by densitometry and normalized with GAPDH. Values are the means ±SEM of at least three independent experiments. Bars with different letters (a-d) are significantly different from one another (*p* < 0.05).

**Figure 2:**
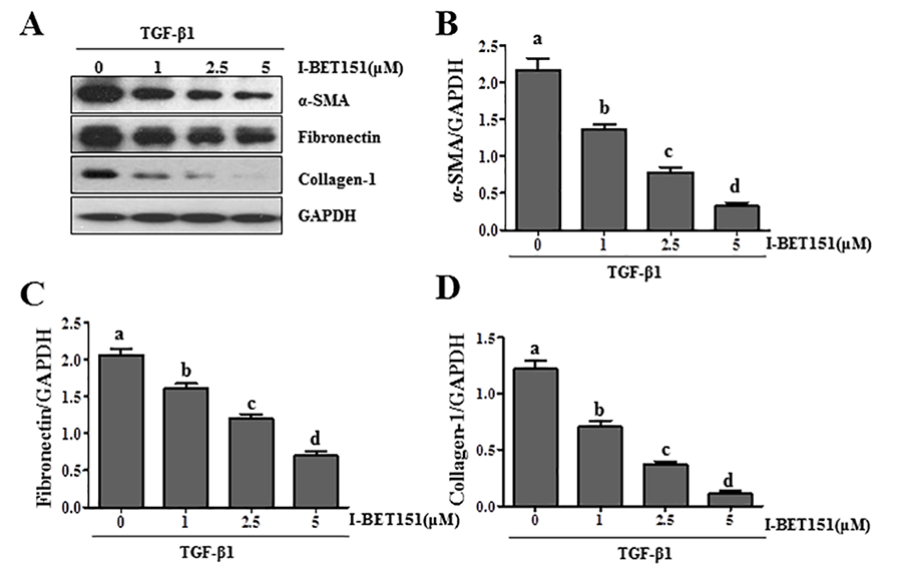
I-BET151 inhibits TGF-β 1-induced activation of renal interstitial fibroblasts in a dose and time dependent manner Normally cultured NRK-49F cells were treated with TGF-β 1 (2 ng/ml) in the presence of I-BET151 (0-5μM) for 36h **A.**. Then, cell lysates were prepared and subjected to immunoblot analysis with antibodies against α-SMA, collagen 1, fibronectin, and GAPDH. The levels of α-SMA **B.**, fibronectin **C.** and collagen-1**D.** were quantified by densitometry and normalized with GAPDH. Values are the means ±SEM of at least three independent experiments. Bars with different letters (a-d) are significantly different from one another (*p* < 0.05).

Activation of renal interstitial fibroblasts is the predominant cellular event indicating the development and progression of renal fibrosis [22, 23]. As a first step towards understanding the role of BET protein in renal fibrosis, we examined the effect of I-BET151on renal fibroblast activation in normally cultured renal interstitial fibroblast cells (NRK-49F) with 5% FBS. As shown in Figure 1A, I-BET151 dose-dependently inhibited the expression of α-smooth muscle actin (α-SMA), the hallmark of fibroblast activation, as well as collagen I and fibronectin, two major ECM proteins. Densitometry analysis of the immunoblot results demonstrated that I-BET151 reduced expression of α-SMA, fibronectin, and collagen 1 by approximately 60%, 70%, and 70, respectively, at a dose of 5 μM (Figure 1B-1D). The time course study with 5μM of I-BET151 (Figure 1E-1H) also showed a significant decrease in the expression level of α-SMA, fibronectin, collagen 1 over time, with a maximum inhibition at 36 hours. Next, we examined the effect of I-BET151 on the TGF-β 1-induced activation of renal fibroblasts. As shown in Figure 2A-2D, I-BET151 also dose-dependently suppressed the TGF-β 1-induced expression of α-SMA, fibronectin and collagen 1. Taken together, these results suggest that BET protein activity is necessary for activation of renal interstitial fibroblasts, and that I-BET151 is a potent inhibitor of BET proteins.

### Silencing of BRD4 inhibits serum and TGF-β 1 induced activation of renal fibroblasts

**Figure 3:**
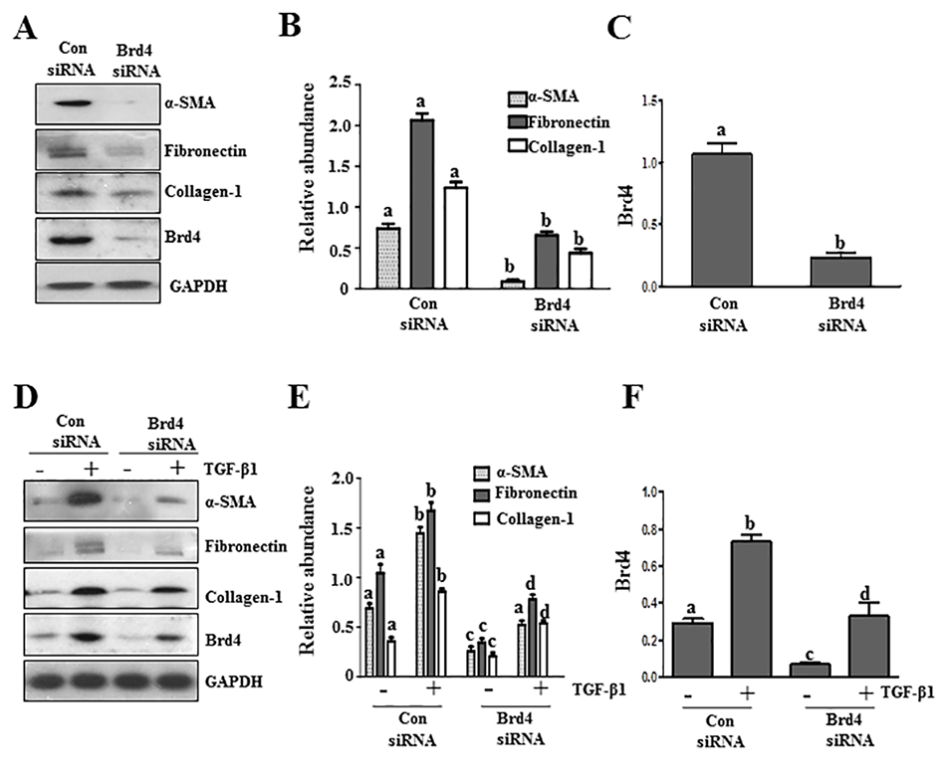
Knockdown of Brd4 with siRNA inhibits fibroblast activation Serum-starved NRK-49F cells were transfected with siRNA targeting Brd4 or scrambled siRNA and then incubated in 5% FBS **A.**-**C.** or TGFβ1 (2 ng/ml) **D.**-**F.** for an additional 24 hours. Cell lysates were prepared for immunoblot analysis with antibodies against α-SMA, fibronectin, collagen1, or GAPDH **A.** and **D.** Expression levels of α-SMA, fibronectin, collagen 1, and Brd4 were quantified by densitometry and normalized with GAPDH **B.**, **C.**, **E.**, and **F.**. Values are the means±SEM of at least three independent experiments. Bars with different letters (a-d) for each molecule are significantly different from each another (*p* < 0.05).

BRD4, a member of the Brd4 family, plays a central role in mediating a variety of biological responses of BET proteins [24, 25].To validate the role of BET proteins in mediating activation of renal fibroblasts, we examined the effect of Brd4 silencing on the expression of α-SMA, fibronectin, and collagen 1 in cultured renal fibroblasts. Our results show that silencing of Brd4 significantly inhibited serum-induced expression of all three aforementioned hallmarks of renal myofibroblasts (Figure 3A, 3B). Silencing of Brd4 also suppressed the TGF-β 1 stimulated expressions of α−SMA, fibronectin, and collagen 1 (Figure 3D, 3E). Brd4 siRNA largely reduced expression of Brd4 (Figure 3A, 3C, 3D, 3F).Thus, these results suggest that Brd4 may be a critical component in the mechanism of BET family protein-mediated renal fibrosis.

### BRD inhibition is protective against renal fibrosis following obstructed injury

**Figure 4:**
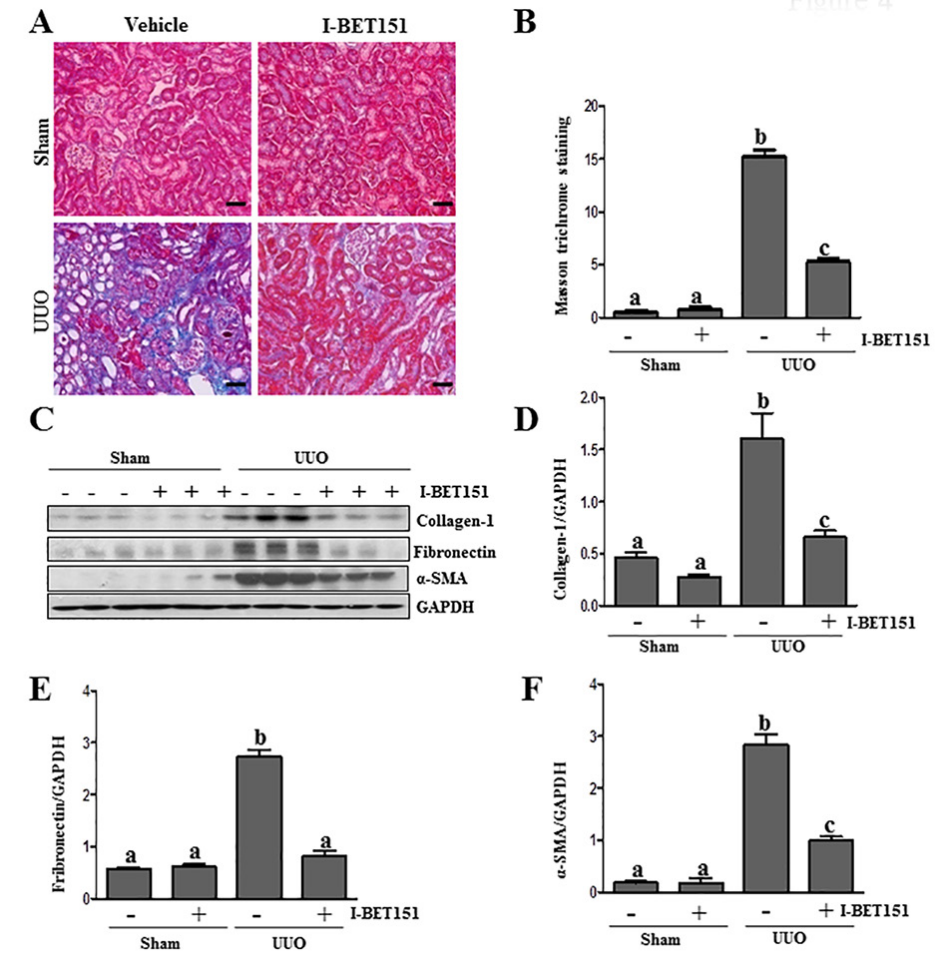
Administration of I-BET151 alleviates development of deposition of ECM in obstructed kidneys **A.** Representative image of Masson trichrome staining of kidney. **B.** The Masson trichrome-positive tubulointerstitial area (blue) relative to the whole area from 10 random cortical fields was evaluated. Data are represented as the mean ± SEM (*n* = 6). **C.** Kidney tissue lysates were subjected to immunoblot analysis with antibodies against collagen-1, α-SMA, fibronectin, or GAPDH. Expression levels of collagen-1, fibronectin, α-SMA, and GAPDH were quantified by densitometry, and the levels of collagen1**D.**, fibronectin **E.**, and α-SMA**F.** were normalized with GAPDH. Values are the means±SEM (*n* = 6). Means with different letters (a-c) are significantly from one another (*P* < 0.05). Scale bar = 50μM.

To assess the role of BET proteins in the development of renal fibrosis, we examined the anti-fibrotic effect of I-BET151 in a murine model of renal fibrosis induced by unilateral ureteral obstruction (UUO). At day 7 following ureteral ligation, kidneys were collected and then subjected to Masson trichrome staining and immunoblots to analyze the expression of ECM proteins. As shown in Figure 4A, collagen fibrils are extensively deposited within the interstitial space after UUO injury, as indicated by an increase in positive areas of Masson trichrome staining. Semi-quantitative analysis of Masson trichrome-positive areas revealed about a 3-fold increase in deposition of ECM components in the obstructed kidney compared with the control kidneys. Administration of I-BET151 reduced ECM deposition by 80% (Figure 4B). To confirm the anti-fibrotic effect of I-BET151, we further examined its effect on the expression of α-SMA and deposition of fibronectin and collagen 1 in obstructed kidneys. Immunoblot analysis of the whole-kidney tissue lysates indicated an increase in the expression of α-SMA, collagen 1, and fibronectin in the kidney after 7 days of UUO injury. Administration of I-BET151 remarkably decreased the level of collagen 1 (~70%) (Figure 4C, 4D, fibronectin (~60%) (Figure 4C, 4E), α-SMA (~60%)(Figure 4C, 4F). Collectively, these data demonstrate that inhibition of BRD by I-BET151 attenuates development of renal fibrosis after UUO injury.

### Administration of I-BET151 reduces Brd 4 level in the kidney following UUO injury

**Figure 5:**
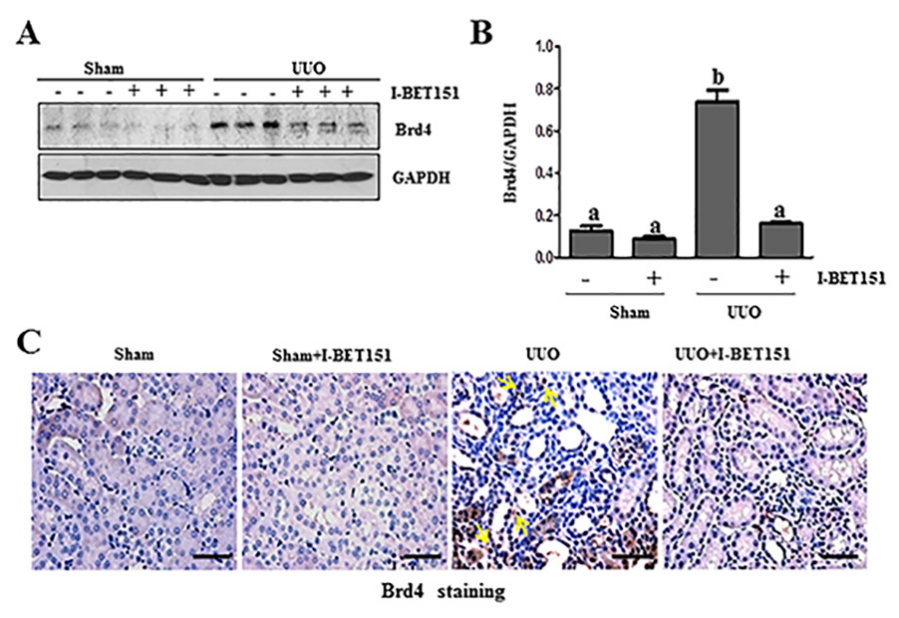
I-BET151 inhibits the expression of Brd4 in obstructed kidneys The prepared tissue lysates from sham operated and obstructed kidneys of mice treated with or without I-BET151 were performed immunoblot analysis with antibodies against Brd4 and GAPDH **A.**. Quantification of Brd4 protein expression was normalized to GAPDH **B.**. Values are the means±SEM (*n* = 6). Means with different letters (a-b) are significantly from one another (*P* < 0.05). **C.** Representative image of Brd4 immunohistochemistry staining of kidney. Arrows indicate interstitial cells. Scale bar = 50μM.

Brd4 has been reported to moderate TGF-β 1-mediated α-SMA induction in lung fibrosis [12]. To demonstrate whether I-BET151 elicited renal fibrosis reduction was associated with the inhibition of Brd4, we performed immunoblot analysis to examine the effect of I-BET151 on Brd4 expression in the murine kidney collected at 7 days after UUO. As shown in Figure 5A-5C, Brd4 was detectable in the kidney of sham-operated mice. UUO injury increased its expression. Administration of I-BET151 reduced the expression of Brd4 to the basal level in the kidney of UUO injured mice. Immunohistochemistry staining also showed increased expression of Brd4 in the kidney after UUO injury, and I-BET151 treatment reduced its expression. Furthermore, we found that Brd4 is expressed in both the renal tubular cells and interstitial myofibroblasts of the injured kidney (Figure 5C). This data suggests that Brd4 may be involved in the regulation of pro-fibrotic machinery in both renal fibroblasts and renal tubular cells.

### BET inhibition suppresses TGF-β/Smad3 signaling

**Figure 6:**
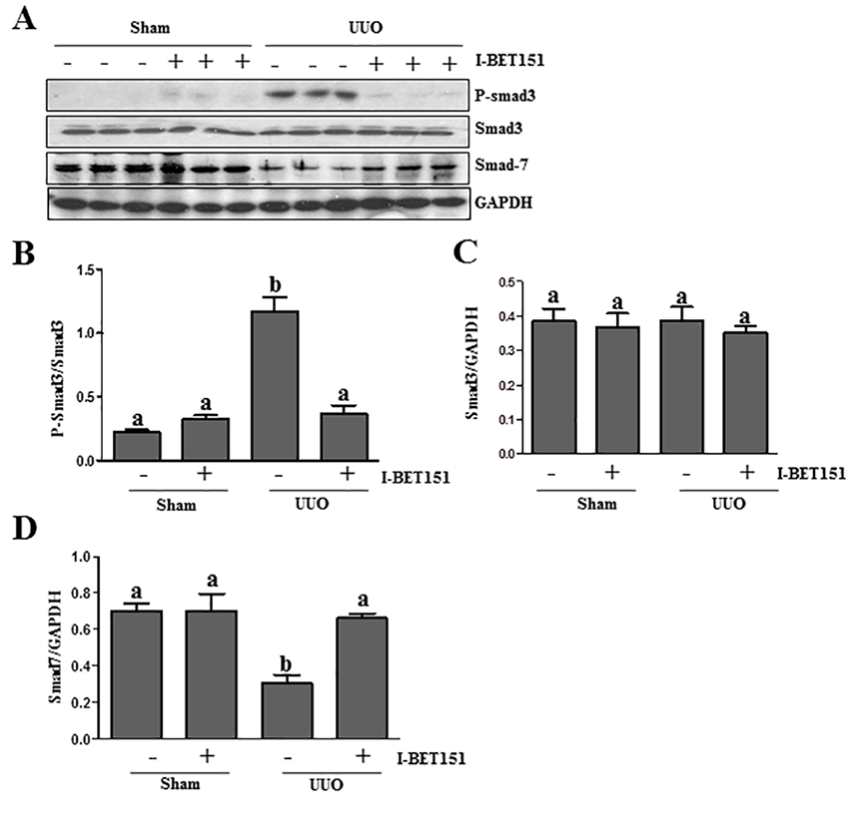
I-BET151 inhibits activation of TGF-β1/Smad3 signalingin UUO models **A.**Kidney tissue lysates were subjected to immunoblot analysis with antibodies to phosopho-Smad3, Smad3, Smad7, or GAPDH. Expression levels of all of those proteins were quantified by densitometry, and Phosopho-Smad3 was normalized to its total protein level **B.**. Smad3 **C.** and Smad7 **D.** levels were normalized to GAPDH. Data are represented as the mean ± SEM. Bars with different letters (a-b) are significantly different from one another (*P* < 0.05).

TGFβ1 is a central cytokine in the regulation of renal fibrosis in various models, which initiates fibrosis *via* the activation of the Smad3 signaling pathway. Conversely, Smad-7 is a key antagonist of TGF-β1 signaling [26–28]. On this basis, we examined the effect of BET inhibition on the phosphorylation of Smad3 and expression of Smad7 in the kidney after ureteral obstruction. As shown in Figure 6A-6D, UUO injury resulted in the phosphorylation of Smad3 and decreased expression levels of Smad7 in the kidney, whereas I-BET151 treatment reduced p-Smad3 expression (Figure 6A) and maintained Smad7 expression levels (Figure 6D). The efficacy of I-BET151 treatment in preserving Smad7 levels may, in part, contribute to the inactivation of TGFβ1 signaling and the attenuation of renal fibrogenesis in the UUO injured kidney.

### I-BET151 inhibits UUO injury-induced EGFR and PDGFRβ phosphorylation

**Figure 7:**
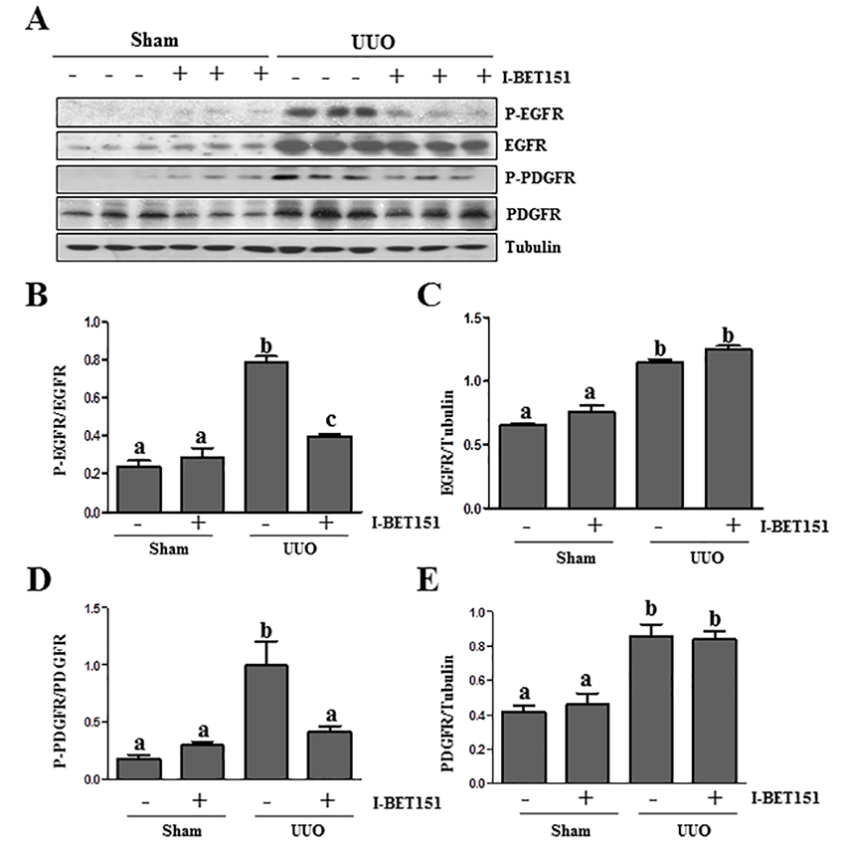
I-BET151 inhibits phosphorylation of EGFR and PDGFRβ in obstructed kidneys **A.** Kidney tissue lysates were subjected to immunoblot analysis with antibodies against phospho-EGFR (Tyr1068), phospho-PDGFRβ(Tyr751), EGFR, PDGFRβ,and GAPDH. All of these proteins were quantified by densitometry, andphospho-EGFR and PDGFRβ**B.** and **D.** were normalized to their total protein levels; EGFR**C.** and PDGFR **E.** were normalized with Tubulin. Data are represented as the mean ± SEM. Means with different superscript letters (a-c) are significantly different from one another (*P* < 0.05).

Results from our previous studies, as well as those of other groups have shown that the activation of EGFR and PDGFRβ plays an important role in renal fibroblast activation and proliferation [29–31]. As such, we further examined the effect of I-BET151on EGFR and PDGFRβ expression and phosphorylation in the kidney by immunoblot analysis. The phosphorylated EGFR at Tyr1068 (p-EGFR) and PDGFRβ at Tyr751 (p-PDGFRβ) were barely detectable in the sham-operated kidneys. After UUO injury, their phosphorylation levels greatly increased in the kidney; however, administration of I-BET151 blocked their phosphorylation (Figure 7A, 7B, 7D). UUO injury also increased total EGFR and PDGFRβ levels, but I-BET151 treatment did not affect their expression (Figure 7A, 7C and 7E). This suggests that BET proteins playa regulatory role in the phosphorylation, but not the expression of these two tyrosine kinase receptors.

### I-BET151 suppresses the phosphorylation and acetylation of NF-κB and STAT3 in the UUO injured kidney

**Figure 8:**
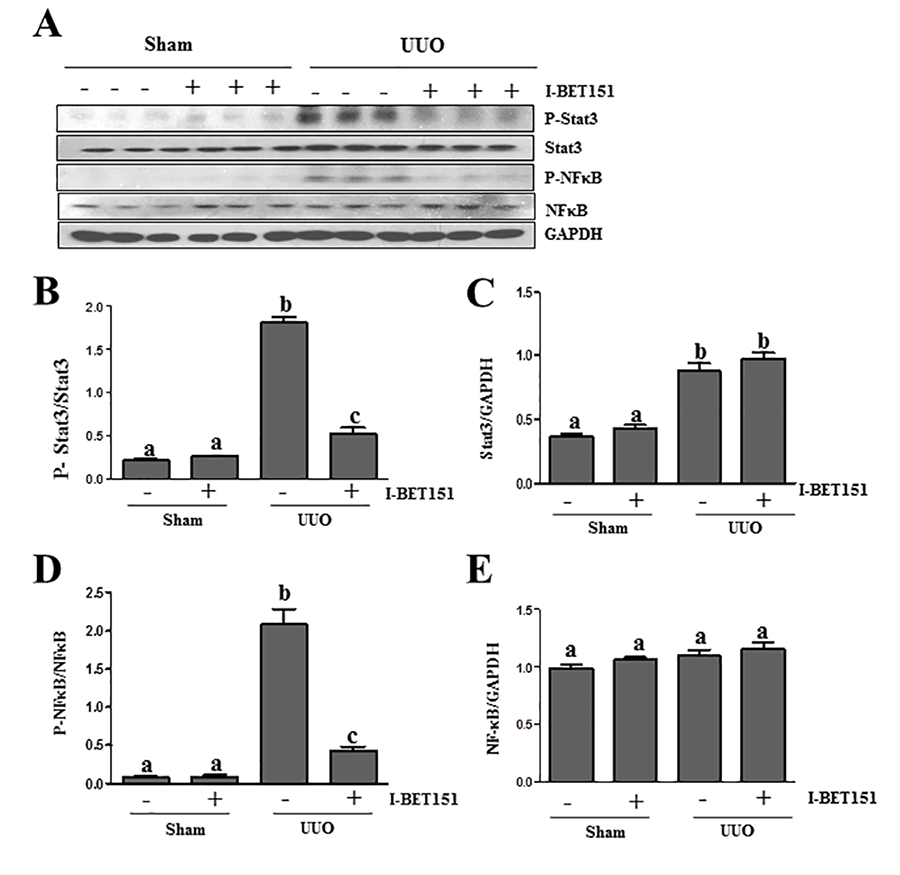
I-BET151 inhibits phosphorylation of STAT3 and NF-κB in the kidney after obstructed injury **A.** Kidney tissue lysates were subjected to immunoblot analysis with specific antibodies against p-STAT3, STAT3, p-NF-κB,NF-κB, or GAPDH. Expression levels of p-STAT3 **B.** and p-NF-κB **D.** were quantified by densitometry and normalized with STAT3 and NF-κB. Expression levels of STAT3 **C.** and NF-κB **E.** were quantified by densitometry and normalized with GADPH. Data are means ± SEM. Means with different superscript letters (a-c) are significantly different from one another (*P* < 0.05).

Previous studies, including our own, have shown that both NF-κB and STAT3 signal pathways are implicated in the pathogenesis of chronic kidney disease [17, 32–34]. Emerging evidence indicates that I-BET151 has a strong inhibitory effect on the activation of NF-κB [35, 36]. To investigate the effect of I-BET151 on the expression, phosphorylation and acetylation of STAT3 and NF-κB in the obstructed kidney, we conducted an immunoblot analysis. I-BET151 inhibited UUO-induced phosphorylation and acetylation of both NF-*κ*B and STAT3 (Figure 8A, 8B, 8D, Supplemental Figure 1). Although expression levels of total STAT3 were also increased in the UUO injured kidney, I-BET151 treatment did not affect their expression (Figure 8A, 8C). UUO injury and I-BET151 had no effect on the expression of total NF-*κ*B. Since activation of both NF-*κ*B and STAT3 are involved in inflammatory responses, it is likely that I-BET151 is able to suppress UUO injury-induced renal inflammation.

In addition, we found that UUO injury resulted in increased phosphorylation of ERK1 and decreased phosphorylation of ERK2, whereas I-BET151 treatment partially inhibited these responses (Supplemental Figure 2). The functional significance of BET-mediated distinct regulation of ERK1/2 needs further investigations.

### I-BET151 suppresses the expression of MCP-1 and infiltration of macrophage cells in the UUO injured kidney

**Figure 9:**
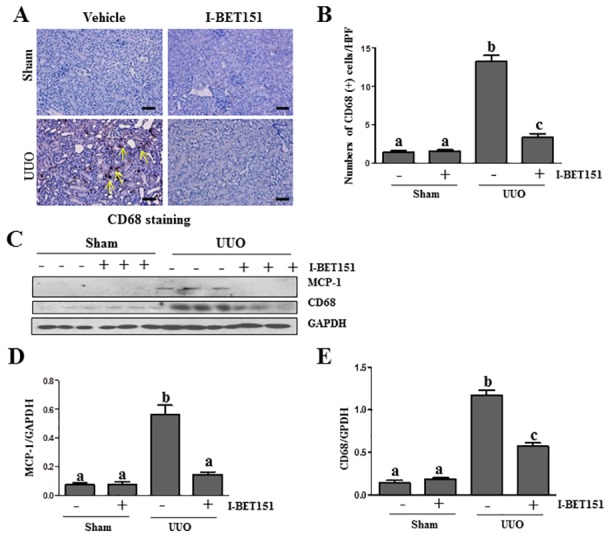
I-BET151 suppresses expression of MCP-1 and infiltration of macrophage in the obstructed kidneys **A.** CD68 (a macrophage marker) was stained with immunohistochemistry. **B.** The corresponding group data show the number of positive staining cells per high power field (HP). **C.** Kidney tissue lysates were subjected to immunoblot analysis with specific antibodies against CD68, MCP-1, or GAPDH. Expression levels ofMCP-1**D.** and CD68 **E.** were quantified by densitometry and normalized with GADPH. Data are means ± SEM. Means with different superscript letters are significantly different from one another (*P* < 0.05). Yellow arrows indicate macrophages.

Inflammation is involved in the progression of chronic kidney disease [37]. Accumulation and infiltration of macrophages in tubulointerstitial areas contributes to pathological alterations of chronic kidney disease [38]. To elucidate the influence of I-BET151 on macrophage infiltration in the kidney after UUO injury, we first examined the expression levels of CD68, a marker of macrophage presence in the kidney *via* immunohistochemistry staining. As shown in Figure 9A, 9B, the CD68 (+) cells were located in tubulointerstitium and increased after UUO injury. I-BET151 treatment completely blocked injury-induced macrophage infiltration. Similarly, I-BET151 abolished CD68 expression in the injured kidney as shown by immunoblot analysis (Figure 9C, 9E). Since monocyte chemoattractant protein-1 (MCP-1) plays a critical role in recruiting macrophages to injured tissues, we also examined the effect of I-BET151 on the expression of MCP-1 in the injured kidney and demonstrated that I-BET151 was effective in decreasing its expression as well (Figure 9C and 9D). These results indicate that I-BET151 is able to inhibit the inflammatory responses in the kidney after UUO injury.

### I-BET151 inhibits UUO injury-induced expression of c-Myc and p53 in the kidney

**Figure 10:**
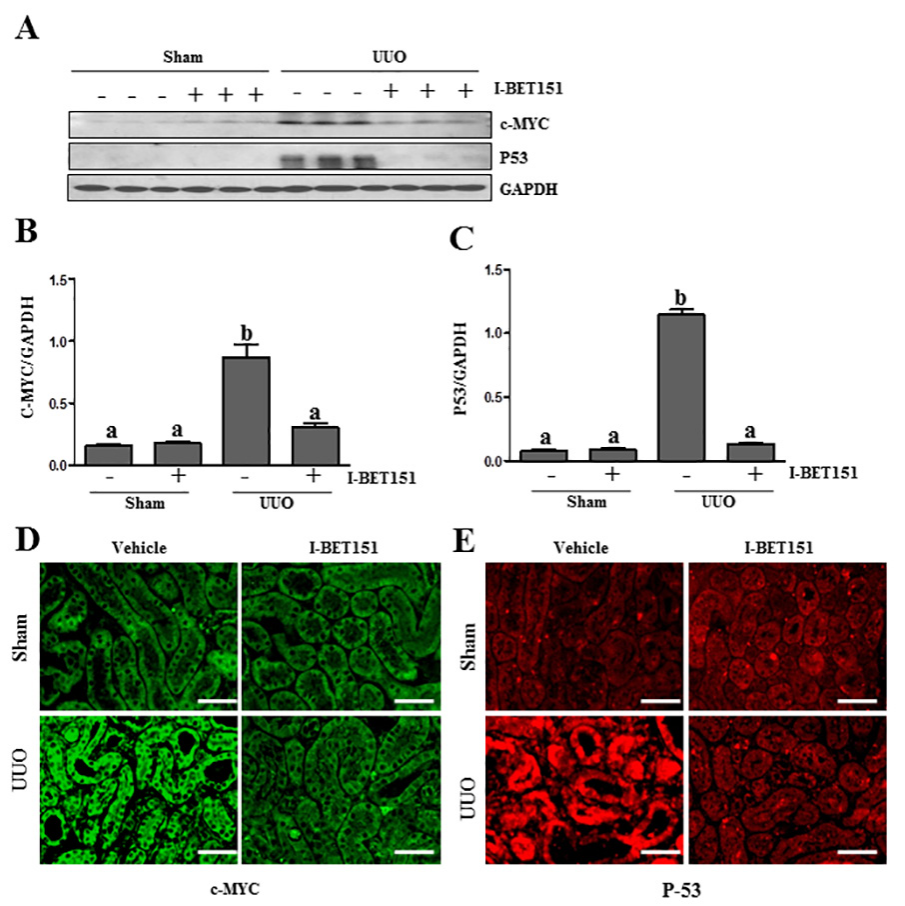
I-BET151 inhibits the expression of c-Myc and p53 in the kidney after obstructed injury **A.** Kidney tissue lysates were subjected to immunoblot analysis with antibodies against c-Myc, p53, and GAPDH. Expression levels of c-Myc **B.** and p53 **C.** were quantified by densitometry and normalized with GADPH. Data are means ± SEM. Means with different superscript letters (a-b) are significantly different from one another (*P* < 0.05). Representative image of c-Myc **D.** and p53 **E.** immunofluorescence staining of kidney. Scale bar = 50μM.

It has been reported that c-Myc and p53 are involved in the regulation of many biological functions including renal fibrosis [39, 40] and that BET proteins are able to regulate their expression [41, 42]. We thus sought out to determine whether BET protein inhibition affects c-Myc and p53 expression in the UUO injured kidneys. As shown in Figure 10, the basal levels of c-Myc and p53 were detected in the sham-treated kidney, and their levels were dramatically increased in the kidney after obstructive injury. Western blot analysis (Figure 10A-10C) indicated that I-BET151 treatment inhibited the expression of c-Myc and p53. We also examined the expression of c-Myc and p53 in the kidney by immunofluorescence analysis. It appears that both of them were mainly expressed in the renal tubules. UUO injury increased their expression and I-BET151 dramatically reduced their expression levels (Figure 10D, 10E). Thus, our data suggests that BET proteins that mediate renal fibrosis may also be associated with activation of c-Myc and p53 transcription factors.

### I-BET151 inhibits renal tubular cells arrested at the G2/M stage of the cell cycle in the UUO injured kidney

**Figure 11:**
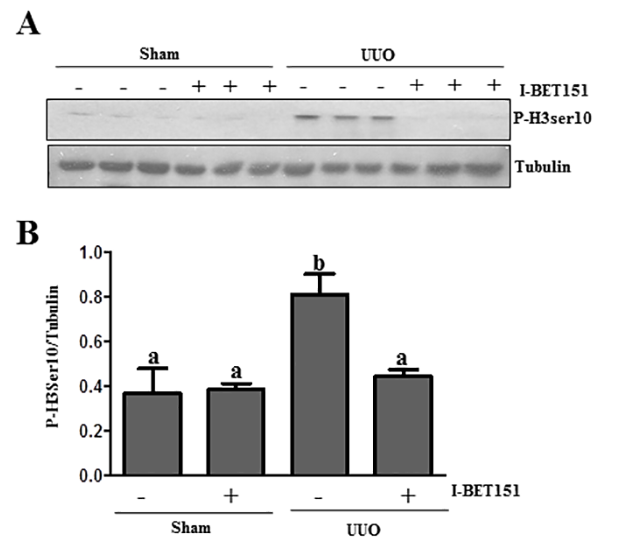
I-BET151 decreases the expression of phospho-histone H3 at Ser10 in the kidney after UUO injury **A.** Kidney tissue lysates were subjected to immunoblot analysis with antibodies against phospho-histone H3 at Ser10 (P-H3ser10) and GAPDH. Expression levels of p-H3 ser10 **B.** were quantified by densitometry and normalized with tubulin. Data are means ± SEM. Bars with different letters (a-b) are significantly different from one another (*P* < 0.05).

Renal epithelial cells arrested at the G2/M phase in the cell cycle resulted in a prominent profibrotic phenotype that produces profibrotic growth factors/cytokines such as TGFβ 1 in the kidney after chronic injury [43]. To determine whether BET proteins mediate this process in the injured kidney, we investigated the effect of I-BET151 on the expression of phospho-histone H3 (ser10) [43], a hallmark of cells arrested at the G2/M stage, in the obstructed kidney by immunoblot analysis. Renal phospho-histone H3 was minimally detectable in sham-operated animals, but significantly increased after UUO injury. Treatment with I-BET151 completely blocked UUO-induced increase of phospho-histone H3 in the kidney (Figure 11A and 11B).Therefore, our data indicate that BET protein inhibition may also attenuate renal fibrosis by reducing the number of renal epithelial cells arrested at the G2/M stage of the cell cycle and subsequently decrease the production of some profibrotic factors.

### Expression of Brd4 in human kidneys with chronic renal diseases

**Figure 12:**
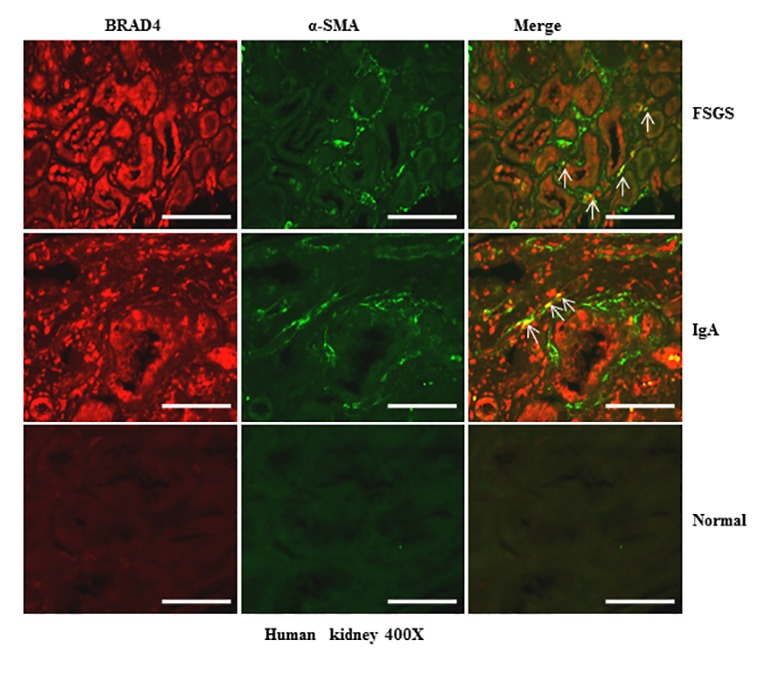
Brd4 is expressed in renal tubular cells and interstitial fibroblasts in human CKD Representative images of double immunofluorescence staining for Brd4 and α-SMA in sections of kidneys biopsies from two random subjects diagnosed with FSGS, IgA nephropathy. Nontumor kidney tissue from patients who had renal cell carcinoma and underwent nephrectomy was used as a control. Arrows indicate Brd4-positive myofibroblasts. Scale bar = 50μM.

To further elucidate the relevance of these results to human disease, we performed immunofluorescence staining to visualize the alteration of Brd4 in the kidney with different diseases and a normal kidney tissue from the normal pole of a kidney resected from a renal tumor. As seen in Figure 12, Brd4 was barely expressed in tubular epithelial cells and α-SMA expression was not detectable in the normal kidney tissue. However, Brd4 was detected in both tubular cells and interstitial cells in the kidney of patients with focal segmental glomerulosclerosis (FSGS) and IgA nephropathy. Notably, some of interstitial cells were co-localized with α-SMA. In line with our findings in the kidney after UUO injury, Brd4 expression also increased in human kidneys with renal diseases. This suggests that Brd4 may play an essential role in mediating the progression of chronic kidney disease.

## DISCUSSION

Compared with extensive studies on BET inhibitors in tumors and other diseases, there are few studies to evaluate the efficacy of BET inhibitors in preclinical models of renal disease. Administration of MS417, a BET specific bromodomain inhibitor, can suppress inflammation and kidney injury in murine models of HIV-associated nephropathy and diabetic nephropathy [44]. JQ1 treatment can also delay cyst growth and kidney enlargement and preserve renal function in early stage genetic mouse strains with PKD1 mutations [45] and ameliorates renal inflammation [13]. In the current study, we extended these observations by examining the effect of BET protein inhibition on renal fibroblast activation and renal fibrosis development after UUO injury. Our results showed that treatment with I-BET151 inhibits activation of renal interstitial fibroblasts and attenuates renal fibrosis *in vivo*. Furthermore, BET inhibition leads to suppression of activation and/or expression of multiple signaling molecules and transcriptional factors associated with renal fibrogenesis. These data indicate that BET bromodomains are critically involved in the activation of profibrotic machinery in the kidney, and suggest that pharmacological targeting of BET proteins could be a therapeutic option for CKD treatment.

Renal fibrosis is the common feature of CKD caused by various etiologies. At the cellular level, activation of renal interstitial fibroblasts is the most important event in renal fibrosis as their persistent activation results in progressive deposition of fibrillar collagens that cause parenchyma damage [46]. Our observations indicate that treatment with I-BET151 inhibited serum and TGF-β 1 induced expression of α-SMA, fibronectin, and collagen 1, in a dose- and time dependent fashion *in vitro* and attenuated renal fibrosis *in vivo*. As serum is the mixture of multiple growth factors and TGF-β 1 is the most potent profibrotic cytokine, diminishing of their effects on renal fibroblast activation by the BET inhibitor underscores the importance of BET bromodomains in mediating activation of the core machinery that propagates myofibroblast activation and renal fibrosis. Indeed, BET inhibition results in inactivation/suppression of multiple molecular and cellular events associated with these processes as listed below.

Firstly, BET inhibition causes inactivation of profibrotic growth factor receptors. Both our own studies and those from other groups have shown that activation of EGFR and PDGFR are important for the activation of renal fibroblasts and the development of renal fibrosis [47, 48]. Here we found that I-BET151treatment dramatically reduced the expression of phosphorylated EGFR and PDGFR in the kidney after UUO injury. The mechanism by which BET proteins regulate phosphorylation of these receptors is not clear at this time. It is possible that BET proteins indirectly regulate activation of these two receptors via modulation of PTEN expression. PTEN is a well-known phosphatase that can induce dephosphorylation of many signaling molecules including EGFR and PDGFR [49, 50]. PTEN can be acetylated and this acetylation inhibits its catalytic activity [51]. Given that BET proteins function as a reader when binding to acetylated proteins, I-BET151 treatment may stabilize PTEN activity through interfering with its acetylation and subsequently inhibit EGFR and PDGFR phosphorylation. Additional studies are needed to further explore the role of PTEN in mediating BET protein regulation of growth factor receptors.

Secondly, BET inhibition blocks activation of multiple intracellular signaling pathways associated with renal fibrosis. Activation of TGF-β1 signaling is central to the development of renal fibrosis in various models. In this pathway, TGF-β1 transduces its fibrotic signal through activation (phosphorylation) of Smad-3. Smad-7, on the other hand, is a key antagonist of TGF-β signaling [26–28]. Thus, we examined the effect of I-BET151 on the phosphorylation of Smad3 and expression of Smad-7 and demonstrated that I-BET151treatment inhibited injury-induced Smad3 phosphorylation and Smad-7 suppression. This suggests that I-BET151 elicited attenuation of renal fibroblast activation is, at least in part, attributed to Smad-7 preservation and subsequent Smad3 inhibition. In addition, we found that I-BET151 inhibits activation of STAT3 and NF-κB, two pathways involved in the regulation of proinflammatory responses, including the production of chemokines and the infiltration of macrophages. As a consequence of their inhibition, we observed that administration of I-BET151 reduced expression of MCP1, a major chemokine in recruiting monocytes to the injured kidney, and CD68, a mark of macrophages in the injured kidney. Therefore, it appears that I-BET151 can attenuate renal fibrosis through suppressing activation of multiple intracellular signaling pathways associated with renal fibroblast activation and proinflammatory responses.

Thirdly, BET inhibition results in suppression of multiple transcription factors. Bromodomain reader proteins bind acetylated histones and recruit transcriptional complexes, therefore representing an important link between histones and transcription. BET inhibitors have been reported to reduce tumor growth through their suppression of multiple transcription factors, including c-Myc and p53 [52, 53]. Since the transcriptional activity of c-Myc and p53 is also associated with induction of many profibrotic genes, we examined the effect of I-BET151 on their expression. Our results showed that I-BET151 treatment significantly suppressed UUO-induced c-Myc and p53 expression in the kidney, suggesting that suppression of c-Myc and p53 may be one of the mechanisms by which I-BET151 inhibits renal fibrosis.

Finally, BET inhibition abrogates the arrest of renal epithelial cells at the G2/M phase of cell cycle, a cellular event associated with the production of profibrotic growth factors/cytokines. After severe renal injury, many tubular cells are reported to arrest in the G2/M phase of the cell cycle and produce profibrotic factors. Subsequently, these factors induce activation and proliferation of pericytes/fibroblasts to accelerate production of extracellular matrix components [54]. Our previous studies have demonstrated that UUO injury can increase the number of renal tubular cells expressing phospho-histone H3 at serine 10, the hallmark of cells at the G2/M phase of the cell cycle [55]. In the current study, we found that Brd 4 is highly expressed in the renal tubule of chronically injured kidneys in mice and humans. These data suggest that BET protein inhibition may block the machinery that induces cell arrest at the G2/M phase. Nevertheless, this may not be the sole mechanism by which BET protein inhibition reduces profibrotic factors. As mentioned above, BET inhibitors-elicited suppression of macrophage infiltration may also contribute to this process.

Currently, the isoform of Brds involved in renal fibrosis remains unclear. It has been reported that Brd4 associates directly with many transcription factors and chromatin-modifying enzymes [56]. Brd4 might therefore be one of the BET proteins that regulate development of renal fibrosis. This was evidenced by our observations that Brd4 is expressed in the kidney injured by ureteral obstruction and from humans with kidney diseases such as FSGS and IgA nephropathy and silencing of BRD4 with its siRNA reduced activation of the cultured renal interstitial fibroblasts. However, since I-BET151 is able to inhibit BRD2, BRD3, and BRD4 activity [9], there remains the possibility that other forms of BET proteins may also be involved in renal fibrogenesis. Further studies are required to identify the I-BET151 target(s) responsible for inhibition of renal fibrosis.

Recently, some BET inhibitors have been shown to have potent antitumor effects in a number of preclinical cancer models [57, 58]. It has also been shown that BET inhibitors confer protection against lung [59] and liver fibrosis [1]. I-BET151 is a highly specific and potent isoxazoloquinoline BET inhibitor, with a pharmacokinetic and bioavailability profile compatible for future clinical development. Like all BET inhibitors, I-BET151 interacts with the Brd pocket in a manner competitive with acetylated peptide binding, leading to the displacement of BET protein from acetylated chromatin in cells exposed to this inhibitor. I-BET151 has been studied in a number of hematological malignancies, including myeloma [21], acute myeloid leukaemia [60], lymphoma [61], and myeloproliferative neoplasms [62]. More recently, I-BET151 showed effective inhibition of osteoclastogenesis and inflammatory bone resorption [63]. Furthermore, clinical trials for three BET inhibitors; BET762, OTX015 and CPI-0610 in refractory haematological malignancies are currently underway. Based on the strong anti-fibrotic effect of I-BET151in the kidney, BET inhibitors could be developed as a potential therapy to treat patients with CKD.

In summary, we were for the first time able to provide evidence that pharmacological targeting of BET proteins can effectively inhibit renal fibroblast activation and attenuate renal fibrosis. Mechanistically, inhibition of BET with I-BET151 led to inactivation of multiple profibrotic signaling pathways, decreased expression of transcription factors, and reduced the number of renal epithelial cells arrested at the G2/M phase. Therefore, BET protein obstruction may represent a novel potential therapeutic approach to inhibit kidney fibrosis after injury.

## MATERIALS AND METHODS

### Chemicals and antibodies

Antibodies to p-STAT3, STAT3, p-Smad3, Smad3, Smad7, p-PDGFR-β, PDGFR-β, p-EGFR (Tyr-845), p-NF-κB, NF-κB, p-53, fibronectin, were purchased from Cell Signaling Technology (Danvers, MA). Antibodies to Brd4, CD68, collagen 1(A2), c-Myc,MCP-1,GAPDH and the immunohistochemistry kit were purchased from Santa Cruz Biotechnology, CA. Small interfering RNA (siRNA) specific for Brd4 and Lipofectamine 2000 were purchased from Invitrogen (Carlsbad, CA). Antibodies to α-SMA and α-tubulin, and all other chemicals were obtained from Sigma (St. Louis, MO).

### Animal model of UUO and treatment protocols

The UUO model was established in male C57BL/6 mice (Jackson Laboratory, Bar Harbor, ME) as described previously [64]. To examine the efficacy of I-BET151 in renal fibrosis after UUO injury, I-BET151 at 30mg/kg in 50 μl DMSO was given *via* I. P. according to previous reports [65, 66]immediately after ureteral ligation based and then administered daily for 7 days before kidneys were harvested. DMSO only-treated animals were used as controls. The animals were killed and the kidneys were collected7 days after surgery. Six mice were used in each group. All animal studies were performed according to the US Guidelines to the Care and Use of Laboratory Animals and approved by the Lifespan Animal Welfare Committee.

### Immunohistochemistry

Kidneys were fixed in formalin and then embedded in paraffin wax. Immunohistochemistry (IHC) was performed to identify macrophages using a rabbit anti-CD68 antibody (1:100) (Santa Cruz) and to determine Brd4 expression using a rabbit anti-Brd4 antibody (1:100) (abcam).Blocking agents included 20% goat serum or donkey serum according to the protocols. Specimens were incubated with the primary antibody overnight at 4°C. immunohistochemistry kit (Santa Cruz Biotechnology, CA) was used for immunoperoxidase staining, and specimens were counterstained with hematoxylin. The specificity of the other antibodies has been established as referenced.

### Histochemical and immunofluorescent staining

To measure renal fibrosis quantitatively, the collagen tissue area was evaluated after Masson trichrome staining using Image Pro-Plus Software (Media-Cybernetics, Silver Spring, MD) by drawing a line around the perimeter of the positive staining area, and calculating and graphing the average ratio to each microscopic field (400 ×). Immunofluorescent staining was carried out according to the procedure described in our previous studies [64]. For immunofluorescent staining, Rabbit anti-P53(1:100), mouse anti-c-Myc (1:100), rabbit anti-Brd4 (1:100) and mouse anti-α-SMA (1:100) (sigma) were used. Fluorescent-conjugated secondary antibodies (1:500) were applied to the sections.

### Cell culture and treatment

NRK-49F cells were purchased from the *ATCC (*Manassas, VA) and cultured in Dulbecco's modified Eagle's medium: Hams F12 medium (DMEM-F12) (Sigma-Aldrich, St Louis, MO) containing 5% fetal bovine serum (FBS), 0.5% penicillin and streptomycin in an atmosphere of 5% CO2 and 95% air at 37 °C. To determine the effects of I-BET151on fibroblast activation, I-BET151 was directly added to subconfluent NRK-49F cells and then incubated for the time as indicated in figure legends. For TGF-β1 treatment, NRK-49F were starved for 24 h by incubation with 0.5% FBS containing DMEM-F12 and then exposed to TGF-β1 (2 ng/ml) for 24 h in the absence or presence of I-BET151.

### Transfection of siRNA into cells

NRK-49F cells were seeded to 50-60% confluence in antibiotic-free medium and grown for 24 h. The siRNA oligonucleotides targeted specifically to Brd4 (100 pmol) were transfected into cells using Lipofectamine (Invitrogen). As a control, 100 pmolof scrambled control siRNA was also transfected into NRK-49Fcells in separate dishes. After transfection, cells were cultured for an additional 48 h in DMEM-F12 with 5% FBS before cell lysates were prepared for immunoblot analysis.

### Western blot analysis

Proteins of kidney lysates were performed by Western blot analysis as described previously [17]. The densitometry analysis of Westernblot results was determined using Image J software (National Institutes of Health, Bethesda, MD).

### Statistical analysis

Data were expressed as means ± SEM for each group. Multiple-group comparison was performed using one-way analysis of variance (ANOVA). Student's test was performed to analyze the differences between two groups. *P* < 0.05 was considered as statistically significant.

## SUPPLEMENTARY MATERIAL


