# Supplementary material for: Pharmacological targeting of BET proteins inhibits renal fibroblast activation and alleviates renal fibrosis
Source: Oncotarget. 2016 Oct 6;7(43):69291–308. doi: 10.18632/oncotarget.12498 (PMC5342478; doi:10.18632/oncotarget.12498)
Supplement: Supplementary file 1 [file oncotarget-07-69291-s001.pdf]

Pharmacological targeting of BET proteins inhibits renal fibroblast activation and alleviates renal fibrosis

Supplementary Material

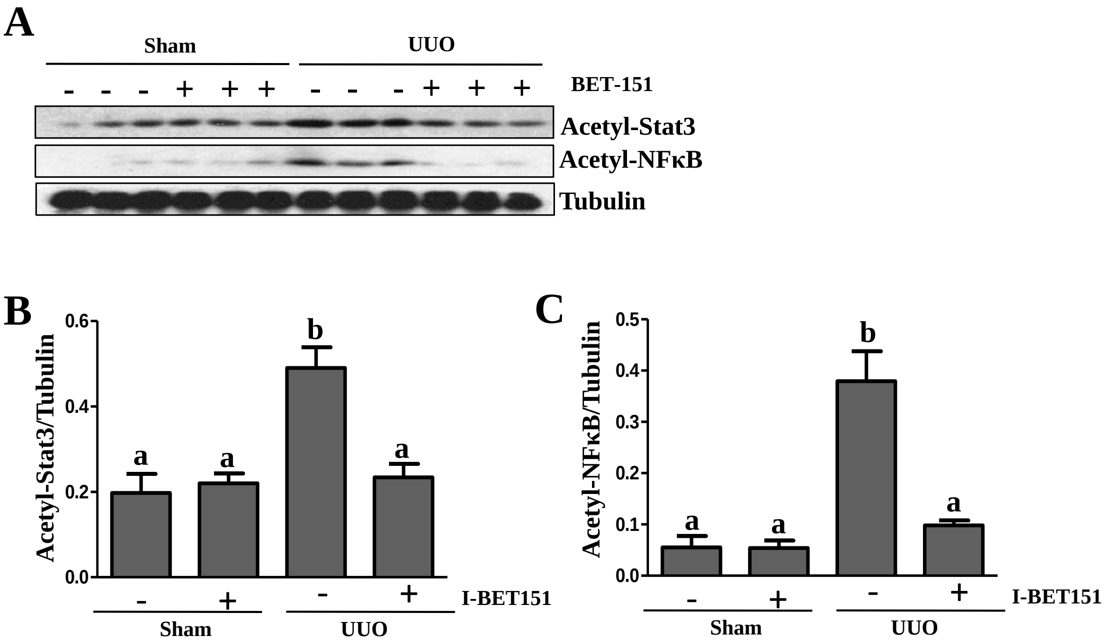

Supplemental Figure 1. I-BET151 inhibits acetylation of STAT3 and NF-κB in the kidney after obstructed injury. (A) Kidney tissue lysates were subjected to immunoblot analysis with specific antibodies against acetyl-STAT3, acetyl-NF-κB or tubulin. Expression levels of acetyl-STAT (B) and acetyl-NF-κB (C) were quantified by densitometry and normalized with tubulin. Data are means ± SEM. Means with different superscript letters (a-b) are significantly different from one another ($P < 0.05$).

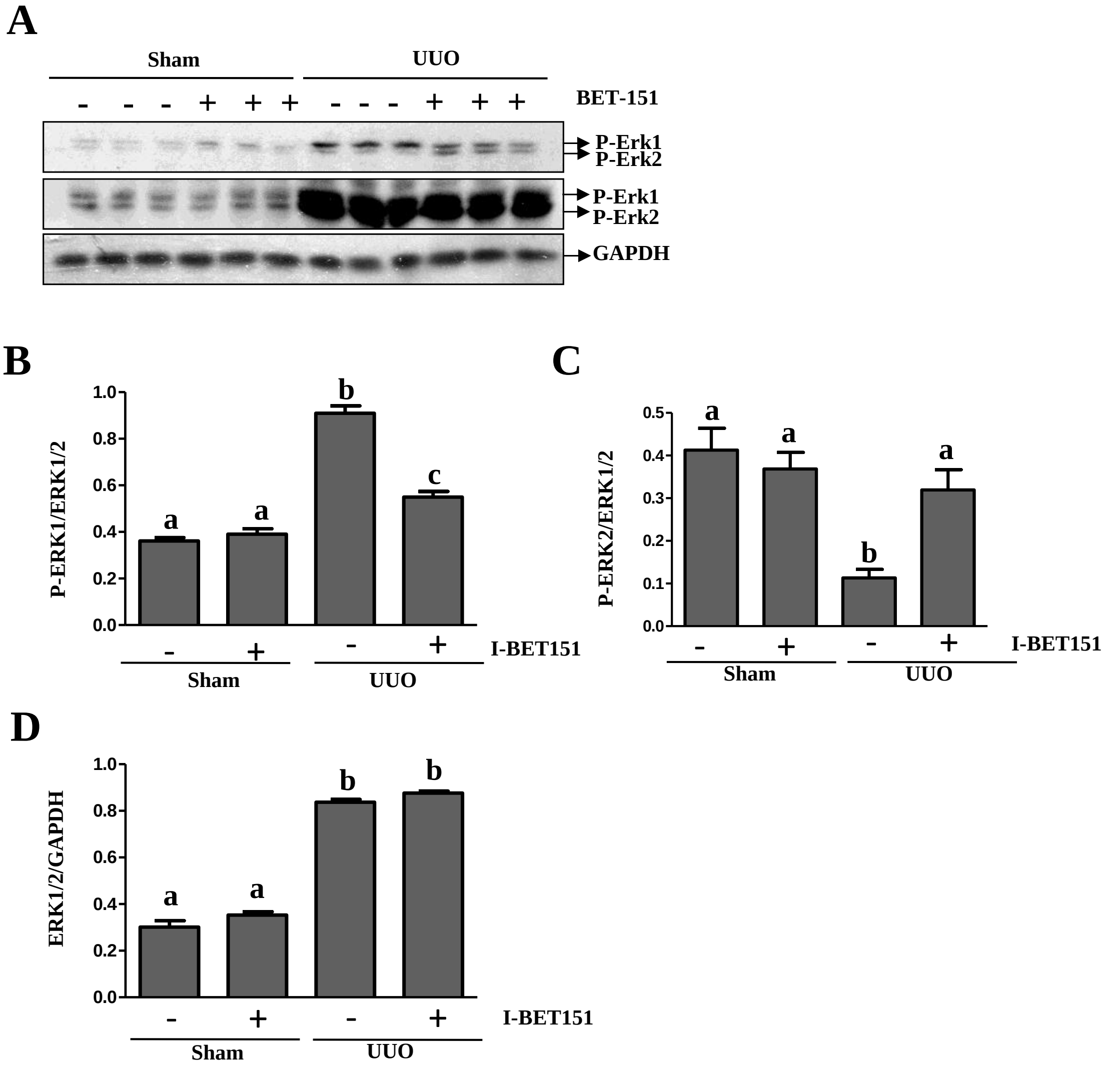

Supplemental Figure 2. Effect of I-BET151 on phosphorylation of Erk1 and Erk2 in the kidney after obstructed injury. (A) Kidney tissue lysates were subjected to immunoblot analysis with specific antibodies against phospho-Erk1/Erk2 and total Erk1/Erk2 or GAPDH. Expression levels of phospho-Erk1 (B) and Phospho-Erk2 (C) were quantified by densitometry and normalized with total Erk1 and Erk2, respectively. Expression levels of total Erk1/2 were normalized with total Erk1/Erk2. Data are means ± SEM. Means with different superscript letters (a-c) are significantly different from one another (*P*< 0.05).
